# Testing a mechanical protocol to replicate impact in walking footwear

**DOI:** 10.1186/1757-1146-7-S1-A68

**Published:** 2014-04-08

**Authors:** Carina Price, Glen Cooper, Philip Graham-Smith, Richard Jones

**Affiliations:** 1 Centre for Health Science Research, University of Salford, Greater Manchester, M6 6PU, UK; 2 School of Engineering, Manchester Metropolitan University, Manchester, UK

## 

**Figure 1:**
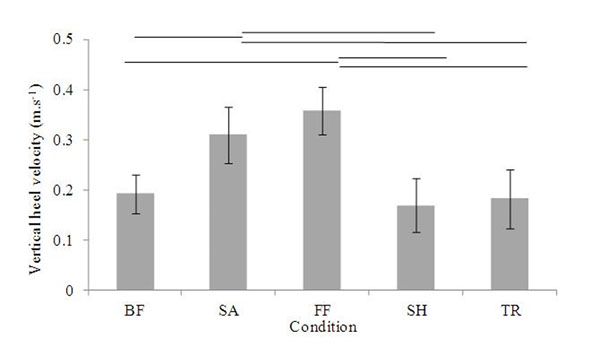
Vertical heel velocity towards the floor in the human testing for the four footwear conditions. Triple-density sandal = SA, flip-flop = FF, shoe = SH and trainer = TR and Barefoot (BF). Error bars denote standard deviation across the 13 subjects tested. Horizontal lines denote statistically significant results (determined by ANOVA where p<.05).

Impact testing is commonly undertaken to quantify the shock absorption characteristics of footwear. The current widely reported mechanical testing method mimics the vertical heel velocity at touchdown and effective mass of the lower limb recorded in running. This therefore results in a greater impact energy than would be expected at touchdown in walking. Despite this mismatch, the methodology is utilised to quantify the shock absorption properties of running and walking footwear alike. The current work modifies the mechanical testing methodology to replicate the kinematics, specifically the vertical heel velocity, identified in walking footwear. Kinematic and kinetic data was collected for 13 subjects walking in four different styles of footwear used for walking (trainer, oxford shoe, flip-flop and triple-density sandal). The kinematic data was utilised to quantify heel velocity at touchdown and accelerometer and force plate data was utilised to estimate the effective mass of the lower limb. When walking in the toe-post style footwear significantly faster vertical heel velocity toward the floor was recorded compared to barefoot and the other footwear styles (Figure 1 for example flip-flop: 0.36±0.05m.s^-1^ compared to trainer: 0.18±0.06m.s^-1^). The mechanical protocol was adapted by altering the mass and drop height from 10.6-17.3 kg and 2-7 mm, compared to the original protocol of 8.45 kg dropped from 50 mm. As expected, the adapted mechanical protocol produced significantly lower peak force and accelerometer values than the ASTM protocol (p <.001). These values more closely resembled those recorded in walking. The mean difference between the human and modified protocol was 12.7±17.5% (p<.001) for peak acceleration and 25.2±17.7% (p=.786) for peak force values. The timing of peak force and acceleration variables was less representative of the real-life data with larger mean differences. This pilot test has demonstrated that the altered mechanical test protocol can more closely replicate loading on the lower limb in walking. Further research should consider more variables related to the shock absorption properties of footwear. The results also demonstrate that testing of material properties of footbeds not only needs to be gait style specific (e.g. running versus walking), but also footwear style specific due to the differences in heel touch-down velocity in footwear styles.

